# Realizing pipe dreams – a detailed picture of vascular development

**DOI:** 10.1093/jxb/erw482

**Published:** 2016-12-22

**Authors:** J. Peter Etchells, Simon R. Turner

**Affiliations:** ^1^Department of Biosciences, Durham University, South Road, Durham DH1 3LE, UK,; ^2^Faculty of Biology, Medicine and Health, University of Manchester, Oxford Road, Manchester M13 9PT, UK

**Keywords:** Cambium, cell division, developmental biology, differentiation, meristems, phloem, vascular development, secondary growth, tissue organization, xylem.

**This special issue of *Journal of Experimental Botany* focuses on the developmental mechanisms required to generate plant vascular tissue. The focus is Arabidopsis, including the three models for initial patterning involving the interaction of auxin and cytokinin, and going on to look at expansion and differentiation into xylem and phloem. Bryophyte and tree models are also considered, as well as new techniques for analyzing the vasculature of mature plants.**

There are many advantages to using plant vascular tissue as a model for studying developmental biology. These include a very high level of tissue organization that is apparent both early in development, where highly coordinated cell divisions in the embryo are required to specify the vascular tissue (Berleth and Jurgens, 1993), and during secondary growth, where highly oriented cell divisions in the cambium, a bifacial meristem, drive radial growth and expansion (Chaffey *et al.*, 2002) (Box 1). Differentiated tissues, the xylem and phloem, are derived from divisions in vascular meristems. Consequently studying vascular tissue formation takes in classic developmental biology themes of cell division, tissue organization and differentiation.

**Box 1. Examples of monocot and dicot vascular tissue**

Monocot vascular tissue such as that of *Sorghum bicolor* (A) is present in bundles (arrowheads) arranged along the radial axis of the stem. Mature vascular bundles are present towards the centre of the stem, while younger bundles are initiated close to the epidermis. Dicot vascular tissue in mature plants is often derived from a continuous ring of cambium (B; arrowhead), such as that found in the Arabidopsis hypocotyl. ph, phloem; x, xylem. Scale bar = 50 μm.

**Figure:**
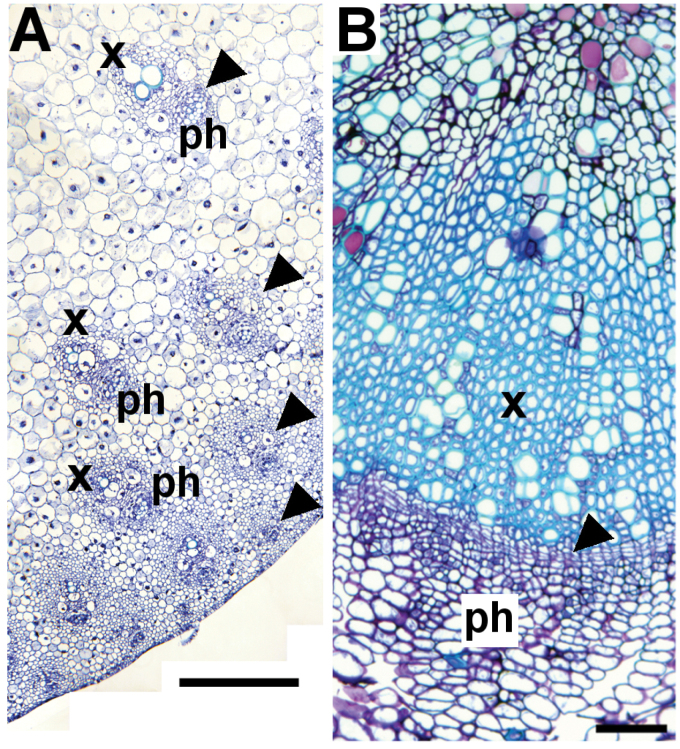

Ohtani *et al.* (2017) review the evolution of water and nutrient transport systems in bryophytes through to the vascular system widely recognizable in higher plants. Terrestrial plants require water and nutrients from the soil and sunlight captured in aboveground organs for growth and development. There would have been considerable selective pressure on early land plants for a transport mechanism that allowed all cells in the plant to access these resources. Vascular systems have enabled plants to increase in size through evolutionary time by facilitating the transport of these resources around the plant and are consequently pivotal to higher plant evolution. Furthermore, innovations such as the rigid secondary cell wall, present in the water-conducting cells of the xylem, enable these vessels to withstand the negative pressures of water transport, but also provide the support allowing increases in plant height to improve light capture. Ohtani *et al.* (2017) also review our knowledge of how transcription factors regulate vascular differentiation, and explore the role these transcription factors may have in driving the evolution of vascular systems in lower plants.

## Pattern specification and expansion

The vascular tissue is specified early in development, and a number of recent papers have focused on how the vascular systems that have evolved in higher plants initiate and expand. In both the embryo and the root of Arabidopsis, a central xylem axis is specified with phloem poles on either side. Several groups have taken a mathematical modelling approach to address the question of how this pattern arises, and in the last couple of years three models have emerged that support the idea that an interaction of auxin and cytokinin probably underpins pattern generation in the root and embryo (De Rybel *et al.*, 2014; Muraro *et al.*, 2014; El-Showk *et al.*, 2015). These models build on our understanding of the roles of auxin and cytokinin and their transport in determining early vascular pattern, gained from genetic and cell biology data over the past decade (Hardtke and Berleth, 1998; Mähönen *et al.*, 2006; Izhaki and Bowman, 2007; Ilegems *et al.*, 2010; Bishopp *et al.*, 2011*a*; Bishopp *et al.*, 2011*b*; De Rybel *et al.*, 2013; Ohashi-Ito *et al.*, 2013). However, the wiring within these models is varied. For example, that put forward by Muraro *et al.* (2014) also incorporates an interaction between the class III homeodomain-leucine zipper (HD-Zip III) transcription factor PHABULOSA (PHB) and cytokinin, an interaction explored in this special issue (Ramachandran *et al.*, 2017). In all cases the models make novel predictions that have subsequently been validated, so certainly all expand our understanding of early vascular development.

How does one compare the differences in the model outputs, and what are the implications of such comparisons? In this special issue, the groups behind the models report further comparisons – simulations have been rerun through each model using the data used to generate the other complementary models (De Rybel *et al.*, 2014; Muraro *et al.*, 2014; El-Showk *et al.*, 2015). There were some disagreements in the outputs, most notably around cytokinin gradients, indicating that further experiments are required. Nevertheless all three models support the interaction of auxin and cytokinin as a plausible system for generating early vascular pattern (Mellor *et al.*, 2017). While these hormone interactions are responsible for generating pattern, they are underpinned by a network of interacting factors, dominated by helix-loop-helix transcription factors, TARGET OF MONOPTEROS 5 (TMO5) (Schlereth *et al.*, 2010), LONESOME HIGHWAY (LHW) (Ohashi-Ito and Bergmann, 2007), SUPRESSOR OF ACAULIS51 (SAC51) (Imai *et al.*, 2006), and SAC51-LIKE (SACL) (Cai *et al.*, 2016), that act downstream of auxin signalling and influence cytokinin activity (reviewed in Campbell and Turner, 2017).

Following specification of the initial pattern, the vasculature rapidly expands to match plant growth. This expansion is underpinned by cell division in the vascular meristems (cambium or procambium), and these cells subsequently differentiate into xylem and phloem. The meristem is maintained by balancing cell division and differentiation. A series of receptor kinases is critical to maintaining this balance, with expression in differing domains of the vasculature. PHLOEM INTERCALATED WITH XYLEM (PXY) is a receptor-like kinase (RLK) that is expressed predominantly on the side of the cambium adjacent to the xylem, and it functions to both promote cell division and repress xylem identity from the cambium (Etchells *et al.*, 2016). The phloem side of the cambium is regulated by a recently characterized RLK, MORE LATERAL GROWTH1 (MOL1), that is related to PXY. In contrast to PXY, MOL1 acts to repress cell division (Gursanscky *et al.*, 2016). A further family of RLKs, members of the ERECTA (ER) family (ERf), also regulate cell division in a number of contexts including the vasculature (Uchida and Tasaka, 2013). *ER* is expressed in the phloem, and it acts redundantly with its closest homologue, *ERL1*, to control vascular proliferation. While the ligand for MOL1 is currently unknown, those for the ERf RLKs *CHALLAH*/*EPIDERMAL PATTERNING FACTOR LIKE6* (*EPFL6*), *CHALLAH-LIKE1* (*CLL1*)/*EPFL5* and *CLL2*/*EPFL4* (Abrash *et al.*, 2011) are expressed in the endodermis (Uchida *et al.*, 2012). The PXY ligand, TDIF, is derived from genes expressed in the phloem. Therefore, differing RLKs mark different vascular domains, as do their respective ligands. These non-cell autonomous relationships and distinct expression domains hint at a role in vascular organization, and these themes of proliferation and organization are explored in depth in Tameshige *et al.* (2017) and Turner and Campbell (2017).

Maintenance of vascular meristems is clearly under tight genetic control. In xylem initials, this differentiation is controlled by a very large gene regulatory network (Taylor-Teeples *et al.*, 2015), in which HD-Zip III genes are prominent (Zhong and Ye, 1999; Baima *et al.*, 2001; Ohashi-Ito *et al.*, 2002; Ochando *et al.*, 2008; Baima *et al.*, 2014; Du *et al.*, 2015). Ramachandran *et al.* (2017) explore the role that HD-Zip III genes play in vascular patterning in many contexts, including in early patterning of the root xylem axis, radial patterning of vascular bundles in the stem, and regulation of cell wall polymer deposition in the xylem. In comparison to xylem specification, less is known about how the phloem cell types are specified and differentiated. However, following identification of *BREVIS RADIX* (*BRX*) (Mouchel *et al.*, 2004) and *OCTOPUS* (*OPT*) (Truernit *et al.*, 2012), genes that control the timing and rate of sieve element differentiation, as well as their regulators, the ligand receptor pair *CLAVATA3/ENDOSPERM SURROUNDING REGION-related 45* (*CLE45*) and *BARELY ANY MERISTEM3* (*BAM3*), a picture is beginning to emerge (Rodriguez-Villalon *et al.*, 2014). In this issue, Otero and Helariutta (2017) focus on companion cell specification, and describe the critical function of these cells in loading nutrients and mobile signals, such as FLOWERING TIME (FT), to neighbouring sieve elements for transport.

## Trees and techniques

Many of the discoveries described above focus on developmental mechanisms characterized in Arabidopsis. While this small annual remains unsurpassed in the plant kingdom as a tool for gene discovery, there remains a challenge in assessing the gene and hormone networks that have been uncovered in it in different contexts. However, a system in which this has been performed effectively is wood deposition in poplar trees. Comparisons between radial growth in the Arabidopsis hypocotyl and in particular poplar trees have been described; however, tree models are also essential for investigating aspects of growth that cannot be addressed in annual plants. Here, Bhalerao and Fischer (2017) describe vascular development from this perspective, reviewing how gene and hormone networks respond during entry into cambial dormancy over winter and exit into growth in spring, and exploring our understanding of the genetic basis of extreme longevity of cambium cells in some trees.

While the advantages of using vascular tissue as a developmental model are mentioned at the beginning of this article, one major drawback to studying vascular tissue – particularly after pattern has been established late in development, during secondary growth – is that rapid radial growth makes live imaging unfeasible. The vascular cell divisions and differentiation process occur many layers into the tissue. Wunderling *et al.* (2017) review recently described methods for analyzing the vasculature in mature plants. Such innovations will enable the generation of more high quality quantitative data, which will be critical in placing new discoveries into context as the field expands.

## Perspectives

All the reviews in this special issue demonstrate that our understanding of the vascular meristem and the differentiation of the xylem and phloem produced from it has improved dramatically. There is, however, no review on vascular development in monocots even though they are by far and away the most important crop species globally. Vascular development in monocots is very distinctive and quite different to that of dicots (Box 1), yet despite their importance to global food security, our understanding of vascular development in these species lags far behind. This is partially a consequence of the fact that much of our current knowledge has been obtained from studying the genetically tractable Arabidopsis, or by analyzing the highly organized arrangement of tissues seen in secondary growth. However, it is still unclear how many of the important genes identified as important in dicot vascular development function in the same way in monocots. Whilst this clearly has the potential to be an interesting study in evolution and development, it is imperative that our understanding of vascular developments in monocots must improve in order to establish whether altering vascular tissue development can contribute to producing better, more resilient crop plants better able to meet the challenges associated with population growth coupled with climate change.
